# Central Administration of Ampelopsin A Isolated from *Vitis vinifera* Ameliorates Cognitive and Memory Function in a Scopolamine-Induced Dementia Model

**DOI:** 10.3390/antiox10060835

**Published:** 2021-05-24

**Authors:** Yuni Hong, Yun-Hyeok Choi, Young-Eun Han, Soo-Jin Oh, Ansoo Lee, Bonggi Lee, Rebecca Magnan, Shi Yong Ryu, Chun Whan Choi, Min Soo Kim

**Affiliations:** 1 Brain Science Institute, Korea Institute of Science and Technology (KIST), Seoul 02792, Korea; yunihong@kist.re.kr (Y.H.); hye6595@kist.re.kr (Y.-E.H.); osj@kist.re.kr (S.-J.O.); alee@kist.re.kr (A.L.); 2 Division of Bio-Medical Science & Technology, KIST School, University of Science and Technology, Seoul 02792, Korea; 3 Natural Product Research Team, Gyeonggi Biocenter, Gyeonggido Business and Science Accelerator, Suwon-si 16229, Korea; choiyh1400@gbsa.or.kr; 4 Convergence Research Center for Dementia, KIST, Seoul 02792, Korea; 5 Department of Food Science and Nutrition, Pukyong National University, Busan 48513, Korea; bong3257@pknu.ac.kr; 6 Department of Neuroscience, Pomona College, Claremont, CA 91711, USA; rmaa2018@mymail.pomona.edu; 7 Korea Research Institute of Chemical Technology, Daejeon 34122, Korea; syryu@krict.re.kr

**Keywords:** ampelopsin A, *Vitis vinifera*, memory behavioral tests, long-term potentiation, CREB/BDNF signals

## Abstract

Neurodegenerative diseases are characterized by the progressive degeneration of the function of the central nervous system or peripheral nervous system and the decline of cognition and memory abilities. The dysfunctions of the cognitive and memory battery are closely related to inhibitions of neurotrophic factor (BDNF) and brain-derived cAMP response element-binding protein (CREB) to associate with the cholinergic system and long-term potentiation. *Vitis vinifera*, the common grapevine, is viewed as the important dietary source of stilbenoids, particularly the widely-studied monomeric resveratrol to be used as a natural compound with wide-ranging therapeutic benefits on neurodegenerative diseases. Here we found that ampelopsin A is a major compound in *V. vinifera* and it has neuroprotective effects on experimental animals. Bath application of ampelopsin A (10 ng/µL) restores the long-term potentiation (LTP) impairment induced by scopolamine (100 μM) in hippocampal CA3-CA1 synapses. Based on these results, we administered the ampelopsin A (10 ng/µL, three times a week) into the third ventricle of the brain in C57BL/6 mice for a month. Chronic administration of ampelopsin A into the brain ameliorated cognitive memory-behaviors in mice given scopolamine (0.8 mg/kg, i.p.). Studies of mice’s hippocampi showed that the response of ampelopsin A was responsible for the restoration of the cholinergic deficits and molecular signal cascades via BDNF/CREB pathways. In conclusion, the central administration of ampelopsin A contributes to increasing neurocognitive and neuroprotective effects on intrinsic neuronal excitability and behaviors, partly through elevated BDNF/CREB-related signaling.

## 1. Introduction

Alzheimer’s disease (AD) is the most common neurodegenerative disease with progressive memory loss and cognitive decline in the elderly [1]. The pathological hallmarks of AD include amyloid-β protein accumulation, tau protein aggregation, excessive oxidative stress, and cholinergic dysfunction [2]. Cholinergic circuits have been implicated in cognitive functioning, especially in hippocampus-dependent memory formation, through the modulation of hippocampal synaptic plasticity and transmission [3]. Several studies revealed that deficits in cholinergic signaling, including cholinergic neurons, acetylcholine (ACh), and its receptors were observed in the brain of AD patients [4]. Thus, acetylcholinesterase (AChE) inhibitors, such as donepezil, have become major therapeutic targets for AD treatment, by increasing the availability of ACh at cholinergic synapses within a short period [5]. However, the benefits of current treatments remain controversial due to their lack of efficacy and critical side-effect for long-term use [6].

Scopolamine is a competitive antagonist of ACh at muscarinic receptors which are the main factors underlying the learning process and memory formation by regulating hippocampal synaptic plasticity [7,8]. The scopolamine-induced memory impairment has been widely used as an experimental animal model for the screening of novel therapeutics in AD [9]. Furthermore, the scopolamine appears to be associated with a significant reduction in the expression of brain-derived neurotrophic factor (BDNF) and cAMP-response element-binding protein (CREB) coupled with BDNF activation in the hippocampus [10]. CREB modulates memory formation, consolidation, and long-term memory persistence by positively controlling BDNF expression in the hippocampus [11,12]. Thus, the CREB/BDNF signaling pathways have been suggested as a potential target for the prevention of AD [13].

*V. vinifera*, the common grapevine, is viewed as the important dietary source of stilbenoids, particularly the widely-studied monomeric resveratrol [14]. Resveratrol has emerged as a natural compound with wide-ranging therapeutic benefits on cancer, cardiovascular, inflammatory, metabolic, and neurodegenerative diseases [15]. Even though resveratrol can be naturally oligomerized to achieve enhanced bioactivity with better potency and selectivity, less attention has been paid to resveratrol oligomers [16,17]. Resveratrol oligomers such as the ampelopsin A have shown promise in the treatment of AD by the interference of neurodegenerative processes, including amyloid cascade, α-synuclein cascade, oxidative damage, and cytotoxicity [18,19,20]. Furthermore, some stilbenoids were assessed for their anti-AChE activity and appeared to be potent AChE inhibitors from natural sources [21,22,23]. Among stilbenoids from extracts of *V. vinifera*, resveratrol and ampelopsin A exhibited more potent anti-amyloidogenic activity than the others [24]. Despite these findings, there is limited evidence evaluating the in vivo neuropharmacological activities of ampelopsin A. Thus, we primarily focused on the anti-amnesic potential of ampelopsin A in the scopolamine-injected mice with memory impairment. The actions of ampelopsin A were further examined at the molecular level by assessing the activity of the cholinergic system as well as the expression of CREB/BDNF signals in the hippocampus.

## 2. Materials and Methods

### 2.1. General Procedures and Plant Material

Proton nuclear magnetic resonance (^1^H-NMR), and carbon nuclear magnetic resonance (^13^C-NMR) spectra were performed on a Bruker (Rheinstetten, Germany) AM 300 NMR spectrometer using TMS as an internal standard. Column chromatography was conducted using a Silica gel 60 (70~230 mesh, Merck KGaA, Darmstadt, Germany), ODS-A (12 nm S-7 μm, YMC GEL, Kyoto, Japan), and Preparative HPLC was performed on LC-8A (Shimadzu, Kyoto, Japan). Thin-layer chromatography analysis was performed on Silica gel 60 F_254_ (Merck KGaA, Darmstadt, Germany) and spots were detected under a UV lamp followed by a 10% H_2_SO_4_ reagent. The stem bark of *V. vinifera* was harvested in October 2020 from the vineyard, Hwaseong-si, Gyeonggido, Korea. A voucher specimen (G095) was deposited at the Bio-center, Gyeonggi Institute of Science and Technology Promotion, Suwon, South Korea.

### 2.2. Spectroscopy of Isolated Ampelopsin A from the Stem Bark of V. vinifera

Brown amorphous powder; ^1^H NMR (300 MHz, acetone-*d*_6_) *δ*: 7.09 (2H, d, *J* = 8.8, H-2′, 6′), 6.88 (2H, d, *J* = 8.3 Hz, H-2, -6), 6.75 (2H, d, *J* = 8.8 Hz, H-3′, 5′), 6.64 (1H, d, *J* = 1.9 Hz, H-14), 6.62 (2H, d, *J* = 8.3 Hz, H-3, 5), 6.42 (1H, d, *J* = 2.5 Hz, H-12′), 6.21 (1H, br s, H-14′), 6.14 (1H, br d, *J* = 1.9 Hz, H-12), 5.45 (1H, d, *J* = 4.9 Hz, H-7), 5.42 (1H, br d, *J* = 4.9 Hz, H-8), 5.42 (1H, d, *J* = 11.3 Hz, H-7′), 4.15 (1H, br d, *J* = 11.3 Hz, H-8′); ^13^C NMR (75 MHz, acetone-*d*_6_) *δ*: 159.5 (C-9), 158.3 (C-11), 158.3 (C-13′), 157.9 (C-2′, 6′), 156.7 (C-11′), 155.5 (C-9), 142.5 (C-9′), 139.8 (C-7), 132.0 (C-13), 130.3 (C-1), 129.3 (C-14), 129.3 (C-3, 5), 128.1 (C-2), 118.3 (C-8), 117.7 (C-10′), 115.4 (C-1′), 115.4 (C-4′), 114.9 (C-3), 109.9 (C-12), 104.9 (C-14′), 100.9 (C-12′), 96.5 (C-10), 87.8 (C-7′), 70.6 (C-6), 48.9 (C-8′), 43.3 (C-5). ESI-MS (positive ion mode): m/z 471 [M + H]^+^.

### 2.3. Slice Preparation and Electrophysiology

Young adult mice (C57BL/6J, age 5–6 weeks) were anesthetized with isoflurane. The brain was quickly removed and immersed in an ice-cold oxygenated high-magnesium artificial cerebral spinal fluid (aCSF) composed of (mM): 130 NaCl, 24 NaHCO_3_, 3.5 KCl, 1.25 NaH_2_PO_4_, 1 CaCl_2_, 3 MgCl_2_, and 10 glucose saturated with 95% O_2_ and 5% CO_2_, at pH 7.4. The brain was attached to the stage of a vibratome (DSK Linear Slicer PRO 7, Dosaka EM, Kyoto, Japan) and 300 μm thickness of transverse slices were cut and recovered in an incubation chamber at room temperature for one hour before recording, in standard oxygenated aCSF composed of (mM): 130 NaCl, 24 NaHCO_3_, 3.5 KCl, 1.25 NaH_2_PO_4_, 1.5 CaCl_2_, 1.5 MgCl_2_, and 10 glucose saturated with 95% O_2_ and 5% CO_2_, at pH 7.4. Slices were placed on the microscope stage and superfused with oxygenated aCSF at room temperature. Whole-cell patch recordings were obtained from CA1 pyramidal neurons in voltage-clamp configuration using a Multiclamp700b (Molecular Devices, Sunnyvale, CA, USA) and a borosilicate patch pipette of 5–7 MΩ resistance. The internal pipette solution for voltage-clamp recordings consisted of (mM): 140 Cs-MeSO_4_, 10 HEPES, 7 NaCl, 4 Mg-ATP, and 0.3 Na_3_-GTP with 1 mM QX314. All neurons included in this study have a resting membrane potential below −55 mV, had an access resistance in 10–20 MΩ, and showed only minimal variation during the recordings. Records were filtered at 2 kHz and digitized at 10 kHz using a Digidata1322A (Molecular Devices, CA, USA). The evoked excitatory postsynaptic current (eEPSCs) was recorded by applying 100 μs current injection (1–200 μA) to a bipolar stimulating electrode placed in the CA1 stratum radiatum of schaffer collateral pathway and analyzed using pCLAMP10 software (Axon Instruments, Burlingame, CA, USA). For LTP recordings, electrical stimulations were given as theta-burst stimulation, consisting of 3 trains containing 4 pulses 15 bursts (each with 4 pulses at 100 Hz) of stimuli delivered every 200 ms.

### 2.4. Animals

Male C57BL/6J mice (8 weeks old; 25–30 g) were purchased from Orient Bio Inc. (Seungnam, Korea). The animals were housed in a room with constant temperature (23 ± 1 °C) and humidity (50 ± 10%) under a 12 h light/dark cycle, and were fed with food and water ad libitum. The experimental procedure was approved by the Institutional Animal Care and Use Committee (IACUC Approval No. KIST-2020-014) and the Institutional Biosafety Committee (IBC), and was conducted in accordance with relevant guidelines and regulation of the IACUC and the IBC in the Korea Institute of Science and Technology (KIST).

### 2.5. Surgical Procedure and Treatments

After one week of acclimatization, mice underwent stereotaxic surgery for implantation of a cannula in the brain as previously described [25]. Using a stereotaxic frame (Kopf Instruments, Tujunga, CA, USA), a 26-gauge guide cannula (Plastics One, Roanoke, VA, USA) was inserted into the third-ventricle (3 V, coordinates: 2.0 mm posterior to the bregma, 5.3 mm below the surface of skull). Following a week recovery period, mice were randomly divided into three groups (*n* = 5 per group): control (Con, PBS as a vehicle), scopolamine + vehicle (Scop + Veh, PBS as a vehicle), and scopolamine+ampelopsin A (Scop + AmpA) pretreatment group. All groups were administrated three times a week with either 0.5 μL of phosphate-buffered saline (PBS, as a vehicle) or 0.5 μL ampelopsin A (10 ng/µL, dissolved in PBS) for one month. One month later, scopolamine + vehicle (Scop + Veh) and scopolamine + ampelopsin A (Scop + AmpA) pretreatment groups received 0.8 mg/kg scopolamine ((-)-scopolamine hydrobromide trihydrate, dissolved in 0.9% saline, i.p.) and the control group was injected with 0.9% saline (i.p.) before each behavioral test. The experimental schedule of chemical administration and behavioral tests is shown in Figure 3a.

### 2.6. Behavioral Tests

All behavioral tests were performed in the behavior testing room. An Anymaze video-tracking system (Stoelting) equipped with a digital camera connected to a computer was used. Following behavioral tests, open field (locomotion), novel object recognition, and passive avoidance were conducted. The study was carried out in compliance with the ARRIVE guidelines. During the behavioral tests, mice were centrally administered PBS or ampelopsin A before every behavioral test, including habituation and test session (60 min before). Subsequently, mice were treated (i.p.) with saline or scopolamine before every behavioral test (30 min before).

#### 2.6.1. Open Field Test

The open-field test (locomotion) was performed as previously described with slight modifications [26]. Specifically, the mouse was located in the center of an open field chamber (40 cm length × 40 cm width × 50 cm height) and was habituated for 20 min. Each mouse was replaced in the same chamber 24 h later. The movements of the mouse were recorded for 10 min and then analyzed via a digital camera connected to the Any-Maze animal tracking system software (Stoelting, Wood Dale, IL, USA). The total distance moved (meters) and the time (seconds) spent in the center/outer of the open field were measured.

#### 2.6.2. Novel Object Recognition Test

The novel object recognition test was performed as described in the previous study [27]. Specifically, the mouse was located in a square arena (40 cm length × 40 cm width × 50 cm height) equipped with a digital camera and was allowed to familiarize with the environment for 10 min before the test. During the first session (familiarization session), two identical objects were put against the center of the opposite wall and the mouse was allowed to explore the objects for 20 min. During the second session (test session), one of the identical objects was replaced by a novel object, and the mouse was allowed to explore the objects for 10 min. In the familiarization session, the mouse contacted with two yellow square-based pyramids(8 cm × 8 cm × 6.5 cm) while in the test session it was with a yellow cube (7 cm × 7 cm × 7 cm) and a yellow square-based pyramid. The amount of time that the mouse spent exploring each object was monitored and analyzed using an ANY-maze video-tracking system (Stoelting, USA). A discrimination index was calculated as (novel − familiar object exploration time)/(novel + familiar object exploration time).

#### 2.6.3. Passive Avoidance Test

The passive avoidance test was performed as previously described [28] using an Avoidance System (B.S Technolab INC., Seoul, Korea). The apparatus (48 cm length × 23 cm width × 28 cm height) consisted of light and dark chambers separated by a gate. On the first day, the mouse was allowed to explore both compartments freely for 10 min. On the following day (training), the mouse was placed in the light compartment and 60 s later the gate was opened. Once the mouse entered the dark compartment, the door was closed and an electrical foot shock (0.3 mA, 3 s) was delivered through the floor. After 24 h (probe trial), the mouse was placed again in the light compartment and then the gate was lifted 60 s later. The step-through latency, or time taken for the mouse to enter the dark compartment, was scored 300 s as the upper limit.

### 2.7. ChAT Activity

The Choline Acetyltransferase (ChAT) activity in the hippocampus was determined using ChAT Activity Assay Kit (Elabscience, Huston, TX, USA) according to the manufacturer’s protocol. Absorbance at 324 nm was measured using a Tecan Infinite 200 microplate reader (Tecan, Männedorf, Switzerland). Enzyme activity was calculated using the following formula: Enzyme activity: (unit/mg protein) = [(ΔA324)/ × 16.6]/(1.98 × 10^−5^ nM^−1^ cm^−1^ × 24)/[protein concentration (mg/mL)]. Protein concentrations were assayed using a Quick Start Bradford Protein Assay kit (Bio-Rad, Hercules, CA, USA).

### 2.8. Ach Level and AChE Activity

The part of the hippocampus was homogenized on ice using RIPA buffer (Merck KGaA, Darmstadt, Germany) and the homogenates were centrifuged at 16,000× *g* for 20 min, then the supernatant was collected to analyze acetylcholine (Ach) level and acetylcholinesterase (AChE) activity using an Amplex Red Ach/AChE Assay Kit (Invitrogen, Waltham, MA, USA) in accordance with the manufacturer’s protocol. Absorbance at 563 nm was measured using a Tecan Infinite 200 microplate reader (Tecan, Männedorf, Switzerland). Hippocampal Ach level and AChE activity were calculated from a standard curve.

### 2.9. Quantitative Real-Time PCR Analysis

The total RNA from the hippocampus tissue was extracted using Trizol reagent (Invitrogen Life Technologies, Waltham, MA, USA) and cDNA synthesized using a SuperScript III First-Strand Synthesis System for RT-PCR (Invitrogen, Waltham, MA, USA) according to the manufacturer’s instructions. Complementary DNA amplification was performed using Power SYBR Green PCR Master Mix kit (Applied Biosystems, Waltham, MA, USA) and primers with the following sequences: *Bdnf* (NM_007540), 5′-TCATACTTCGGTTGCATGAAGG-3′ and 5′-AGACCTCTCGAACCTGCCC-3′; *TrkB* (NM_001025074), 5′-CTGGGGCTTATGCCTGCTG-3′ and 5′-AGGCTCAGTACACCAAATCCTA-3′; *Akt1* (NM_001165894), 5′-ATGAACGACGTAGCCATTGTG-3′ and 5′-TTGTAGCCAATAAAGGTGCCAT-3′; *Creb1* (NM_009952), 5′-AGCAGCTCATGCAACATCATC-3′ and 5′-AGTCCTTACAGGAAGACTGAACT-3′; *iNOS* (NM_010927), 5′-GGCAGCCTGTGAGACCTTTG-3′ and 5′-TGCATTGGAAGTGAAGCGTTT-3′; *Chrm1* (NM_001112697), 5′-AGTGGCATTCATCGGGATCA-3′ and 5′-CTTGAGCTCTGTGTTGACCTTGA-3′; *Ache* (NM_009599), 5′-AGAAAATATTGCAGCCTTTG-3′ and 5′-CTGCAGGTCTTGAAAATCTC-3′; *CaMK2* (NM_177407), 5′-GAATCTGCCGTCTCTTGAA-3′ and 5′-TCTCTTGCCACTATGTCTTC-3′; *Bcl2* (NM_177410), 5′-AGCTGCACCTGACGCCCTT-3′ and 5′-GTTCAGGTACTCAGTCATCCAC-3′; *Bax* (NM_007527),5′-CGGCGAATTGGAGATGAACTG-3′ and 5′-GCAAAGTAGAAGAGGGCAACC-3′; *Actb* (NM_007393), 5′-GGCTGTATTCCCCTCCATCG-3′ and 5′-CCAGTTGGTAACAATGCCATGT-3′. The StepOne Real-Time PCR System (Applied Biosystems, Waltham, MA, USA) for quantitative PCR (qPCR) was used for quantitative real-time PCR. PCR results were normalized to those of the control genes encoding β-actin (*Actb*).

### 2.10. Western Blot Analysis

The part of the hippocampus was homogenized on ice using RIPA buffer (Sigma, Germany) and the homogenates were centrifuged at 16,000× *g* for 20 min, then the supernatant was collected. The protein concentration was determined as mentioned above. 30 µg of proteins were separated by 10% polyacrylamide gel electrophoresis and transferred to PVDF membranes (Millipore, Burlington, MA, USA). After blocking in 5% skim milk, the membrane was incubated with rabbit anti-BDNF (1:800, Abcam, Cambridge, UK), rabbit anti-phospho CREB (pCREB; 1:2000, Abcam, Cambridge, UK), mouse anti-CREB (1:1000, Invitrogen, Waltham, MA, USA), and mouse anti- β-actin (1:1000, Cell Signaling Technology, Danvers, MA, USA) overnight at 4 °C. The membranes were washed and incubated for 1 h with HRP conjugated anti-rabbit (Abcam, Cambridge, UK) or anti-mouse IgG antibody (Enzo Life Sciences, Farmingdale, NY, USA). The bands were visualized using Image Quant LAS 4000 (GE Healthcare, Chicago, IL, USA) with ECL reagent (Amersham, Little Chalfont, UK), and the intensity was quantified using Image J software (National Institutes of Health, Bethesda, MD, USA).

### 2.11. Statistical Analysis

Experimental values were shown as mean ± standard error of the mean (S.E.M.) and evaluated with one-way ANOVA followed by Dunnett’s test. The statistical analysis was performed using the GraphPad PRISM software (GraphPad Prism Software Inc., version 8, San Diego, CA, USA). *p*-values of <0.05 were deemed significant.

## 3. Results

### 3.1. Isolation and Determination of Compound from the Stem Bark of V. vinifera

**Figure 1:**
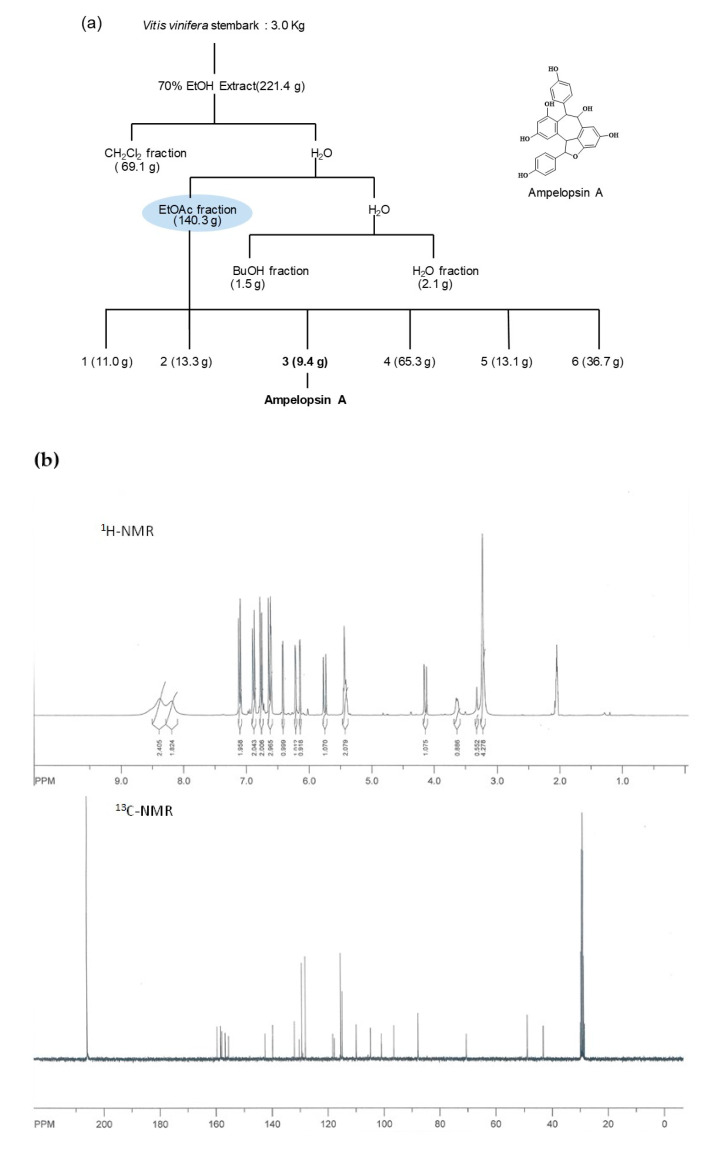

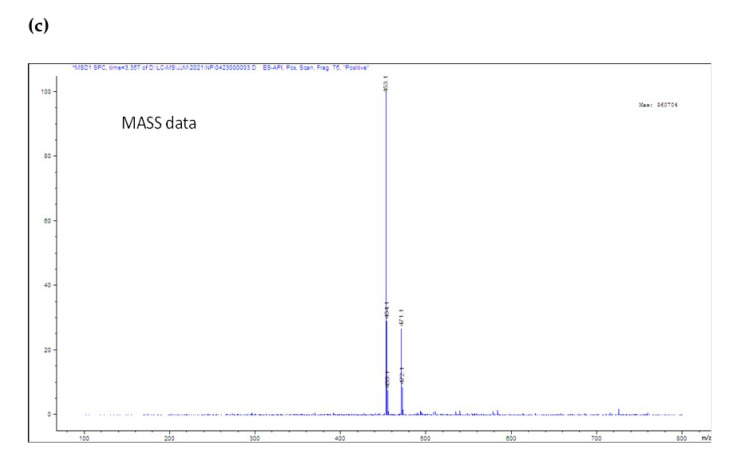
Isolation and structural analysis of ampelopsin A isolated from *V. vinifera*. (**a**) Extraction and purification procedures of ampelopsin A from stembark of *V. vinifera* and its molecular structure. (**b**) ^1^H-NMR and ^13^C-NMR (300 MHz, acetone-*d*_6_) spectrums of ampelopsin A from stembark of *V. vinifera*, (**c**) Mass spectrum of ampelopsin A from stembark of *V. vinifera*.

The stem bark of *V. vinifera* (3.0 kg) was extracted twice with 15 L of 70% ethanol (EtOH) by two times at room temperature (each time for 2 days). After filtration with a cotton ball, the filtrate was combined and evaporated to dryness to give 221.4 g of dark syrupy extract. The extracts were suspended in distilled water and then partitioned CH_2_Cl_2_ (5.0 L × 3), EtOAc (5.0 L × 3), and n-butanol (5.0 L × 3) to give CH_2_Cl_2_ (69.1 g), EtOAc (140.3 g), n-butanol (1.5 g), and water-soluble fractions (2.1 g), (Figure 1a). The EtOAc soluble fraction was subjected to silica gel (2.0 kg) column (10 × 60 cm) chromatography, eluted with MeOH in CH_2_Cl_2_ in a step-gradient manner from 1% to 50% to give six fractions (F1: 11.0 g, F2: 13.3 g, F3: 9.4 g, F4: 65.3 g, F5: 13.1 g, and F6: 36.7 g). Fraction F3 (9.4 g) was separated by MPLC chromatography that used gradient mixtures as eluents (F31–F38). F34 was also purified in a similar manner with RP-18 preparative HPLC eluted with MeOH in H_2_O (1% to 100%) in a stepwise gradient, which finally gave 8.2 mg of compound (Purity 97% in HPLC). The molecular formula of the compound was confirmed by Mass spectrum as C_28_H_20_O_7_, consisting of 19 degrees of unsaturation (Figure 1c). The ^1^H-NMR spectrum of the compound indicated five pairs of peaks. Two pairs appeared at δ 6.88/6.62 and 7.09/6.75, each peak with characteristic *ortho* and *meta* couplings; Each peak has an integral value of two. They were assigned to the protons of two *para*-disubstituted aromatic rings, A and A′. Two other pairs resonating at δ6.64/6.14 and 6.42/6.21 were assigned as meta protons on two tetra substituted aromatic rings, B and B′ (Figure 1b). With the above data and comparison with literature [29], the compound was identified as ampelopsin A.

### 3.2. Bath Application of Ampelopsin A Increases the Neuronal Excitability of Hippocampal Neurons

**Figure 2:**
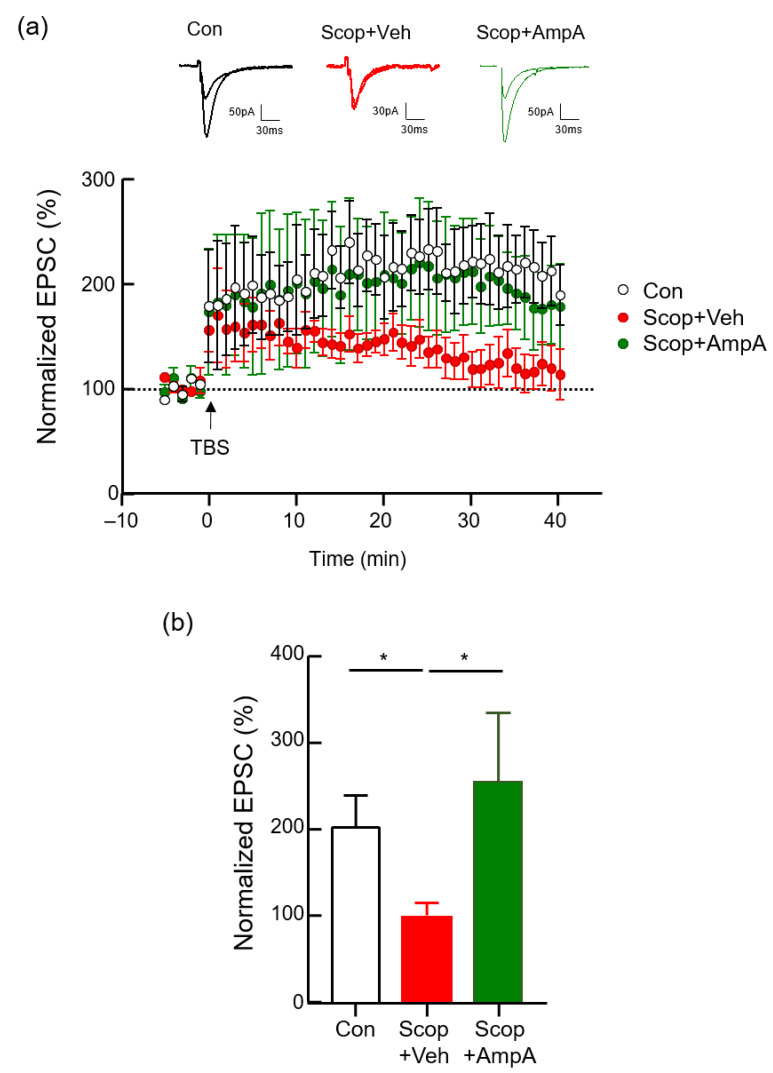
Protective effect of ampelopsin A in hippocampal LTP. A change in the EPSC slope was monitored following LTP induction by theta-burst stimulation (TBS) at SC-CA1 synapses in the hippocampus. The magnitude of LTP was quantified as an increase in the EPSC amplitude relative to the baseline. (**a**) Averaged traces of normalized EPSC amplitude in control, scopolamine (100 μM) with DMSO vehicle, and scopolamine with ampelopsin A (10 ng/µL) group (scale bars, 30 pA or 50 pA, 30 ms). Scopolamine with DMSO or ampelopsin A was treated during the baseline recording and for an additional 20 min after LTP induction by bath application. (**b**) Bar graph of the means for the normalized EPSC amplitude recorded last 5 min, calculated from the data in Figure 2a. Values are expressed as means ± SEM (*n* = 4). * *p* < 0.05; one-way ANOVA with Kruskal-Wallis test with Dunnett’s multiple comparisons test.

Long-term potentiation (LTP) is a long-lasting increase of postsynaptic responses following electrical stimulation such as theta-burst stimulation (TBS) or a brief, high-frequency stimulation (HFS), leading to an enhancement in the strength of excitatory synaptic transmission and it is considered the generally studied form for examining the synaptic mechanism of learning and memory in the brain [30,31]. We examined whether the bath application of ampelopsin A to the brain rescues the scopolamine-induced deficit in hippocampal LTP. For the measurement of hippocampal LTP, a single dose of ampelopsin A (10 ng/µL) was applied to the hippocampal slices which were perfused with artificial cerebrospinal fluid (aCSF) containing either DMSO vehicle or scopolamine (100 μM) during the baseline recording and for an additional 20 min after LTP induction. In control mice, TBS (consisting of 3 trains containing 4 pulses 15 bursts) induces a robust increase in the percentage of normalized excitatory postsynaptic current (EPSC) (Figure 2a). Scopolamine treatment markedly decreased the mean of normalized EPSC relative to the control group (Figure 2b, *p* < 0.05). In the scopolamine and ampelopsin A combined treatment group, the mean of normalized EPSC significantly increased relative to the scopolamine only treatment group (*p* < 0.05).

### 3.3. Administration of Ampelopsin A into the 3 V Increased Cognitive Memory Behaviors

**Figure 3:**
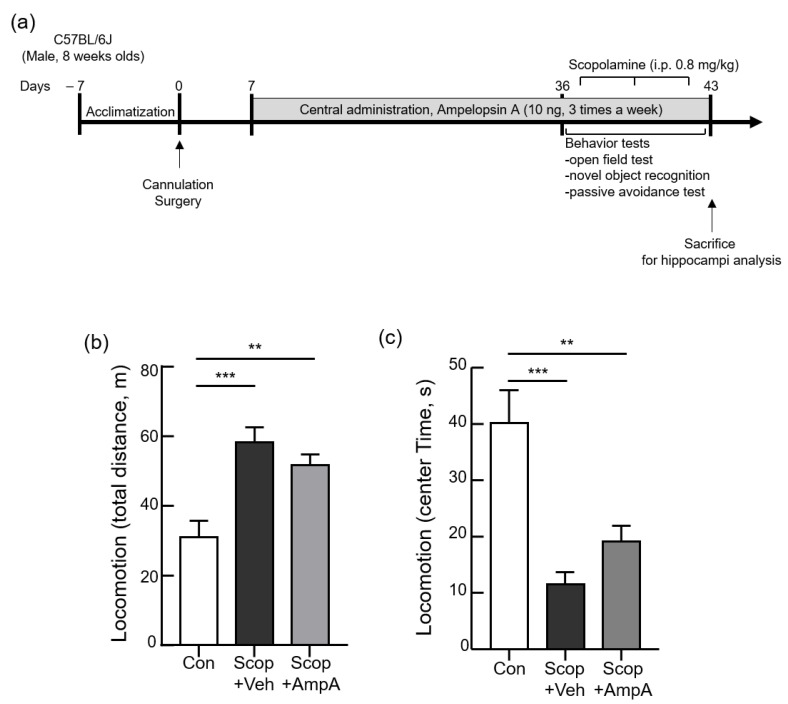
Central administration of ampelopsin A changes the locomotion. Male C57BL/6J mice (10 weeks old) were given 0.5 µL ampelopsin A (10 ng/µL, three times a week) or the same volume of the vehicle (PBS) via the third-ventricle of the brain for one month. 30 min before the behavioral tests, scopolamine (0.8 mg/kg, i.p.) was administered to each group; Scop + Veh (pre-treatment of PBS as a vehicle, scopolamine injection on the experimental day) and Scop + AmpA (pre-treatment of ampelopsin A, scopolamine injection on the experimental day) groups. The control group (Con) was pre-treated with the vehicle into 3 V then was injected PBS (i.p.) instead of scopolamine on the experimental day. (**a**) The schematic timeline of the experiments. (**b**) Total distance and (**c**) time spent exploring the center zone were measured by an open field test (locomotion). Values are expressed as means ± SEM (*n* = 5). ** *p* < 0.01, *** *p* < 0.001; one-way ANOVA with Dunnett’s multiple comparisons test.

Based on the rescue effects of ampelopin A in EPSC measurements, we tried to figure out whether central administration of ampleopsin A restores cognition and memory function of the animals which are impaired by scopolamine injection. We designed the experimental schedules (Figure 3a) and conducted stereotaxic surgery on animals which were implanted cannulas into the third ventricle of the brain. We applied the same dose of ampleopsin A (10 ng/µL) via a cannula into the third ventricle of the experimental animals for one month while other animals were applied the PBS as a vehicle in the same way. After this pre-treatment of ampelopsin A (AmpA) over one month, we injected scopolamine (Scop, 0.8 mg/kg, i.p.) into Scop + Veh (pre-treatments of a vehicle then injected the scopolamine) and Scop + AmpA (pre-treatment of ampelopsin A then injected the scopolamine) group before each behavioral test (30 min before).

#### 3.3.1. Open Field Test

An open field test performed before other behavioral analyses ensured that scopolamine worked properly by using its anxiogenic [32] and locomotor stimulant properties [33]. In the open field test, the scopolamine-injected group (Scop + Veh) showed significantly increased total distance traveled by the mice and shortened the time spent in the center zone compared with the control group (Figure 3b, F_(2,12)_ = 12.64 and Figure 3c, F_(2,12)_ = 14.71, *p* < 0.001). In addition, no significant differences were observed between the Scop + Veh group and Scop + AmpA group. It reveals that ampelopsin A was not associated with anxiety and hyper locomotion caused by scopolamine.

#### 3.3.2. Novel Object Recognition Test

**Figure 4:**
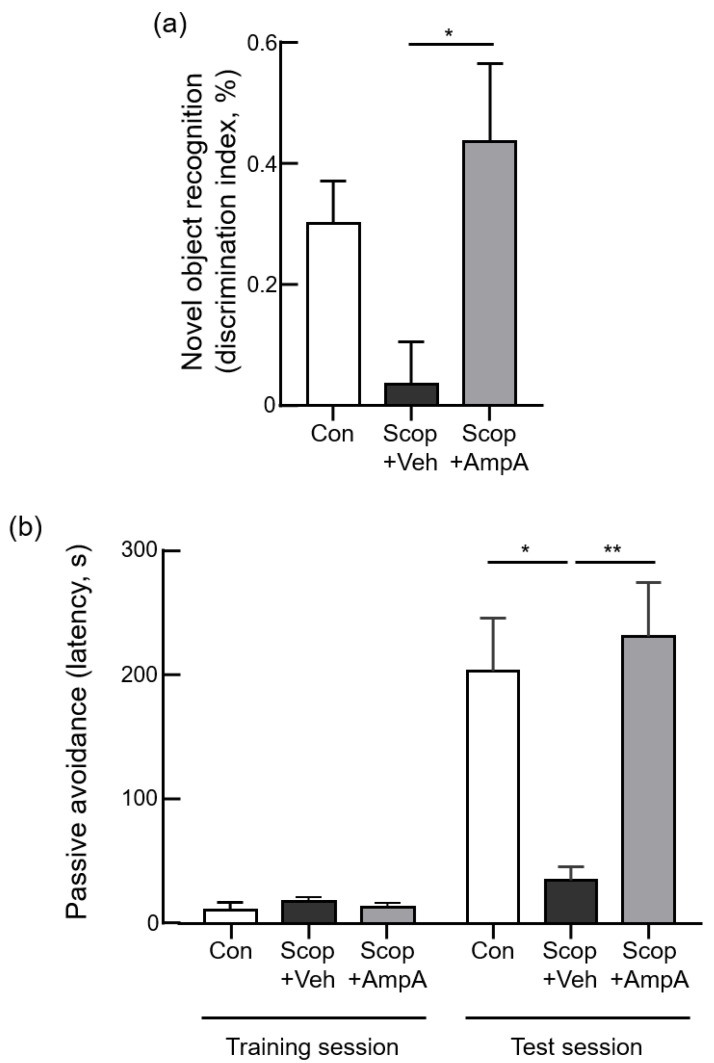
Central administration of ampelopsin A improves cognitive memory behaviors. Male C57BL/6J mice (10 weeks old) were given 0.5 µL ampelopsin A (10 ng/µL, three times a week) or the same volume of the vehicle (PBS) via the third-ventricle of the brain for one month. 30 min before the behavioral tests, scopolamine (0.8 mg/kg, i.p.) was administered to each group; Scop + Veh (pre-treatment of PBS as a vehicle, scopolamine injection on the experimental day) and Scop + AmpA (pre-treatment of ampelopsin A, scopolamine injection on the experimental day) groups. The control group (Con) was pre-treated with the vehicle into 3 V then was injected PBS (i.p.) instead of scopolamine on the experimental day. In the novel object recognition test (**a**), the discrimination index showed the percent time spent with the novel object. In the passive avoidance test (**b**), mice were trained that once the mouse entered the dark compartment, the door was closed, and an electrical foot shock (0.3 mA, 3 s) was delivered through the floor (training session). After 24 h, the moving time to a darkened chamber in a shock-motivated was recorded as a latency time in the test session. Values are expressed as means ± SEM (*n* = 5). * *p* < 0.05, ** *p* < 0.01; one-way ANOVA with Dunnett’s multiple comparisons test.

We conducted a novel object recognition test and passive avoidance test to confirm the restoration of cognition and memory abilities by the administration of ampelopsin Ain memory-impaired models. The time spent with the novel object divided by the total time devoted to exploring both objects, expressed as the discrimination index, was shortened in the Scop + Veh groups than the control group (Figure 4a). However, the Scop + AmpA group markedly increased the discrimination index, indicating that ampelopsin A treatment ameliorated scopolamine-induced recognition memory impairment (F_(2,12)_ = 4.811, *p* < 0.05).

#### 3.3.3. Passive Avoidance Test

In the step-through passive avoidance test, during the training trial, step-through latency was statistically the same amongst all the groups (Figure 4b, F_(2,12)_ = 0.8304, *p* = 0.459). The Scop + Veh group showed a significant decrease in step-through latency in comparison with the control group (F_(2,12)_ = 8.671, *p* < 0.005). A significant increase of step-through latency was presented in the Scop + AmpA group, suggesting that ampelopsin A recovered scopolamine-induced memory impairment in the experimental animals. Interestingly, the Scop + AmpA group showed similar levels with the control group. It means that scopolamine impairments did not work on memory dysfunction by chronic treatments of ampelopsin A.

### 3.4. Administration of Ampelopsin A Ameliorates Cholinergic Dysfunction

**Figure 5:**
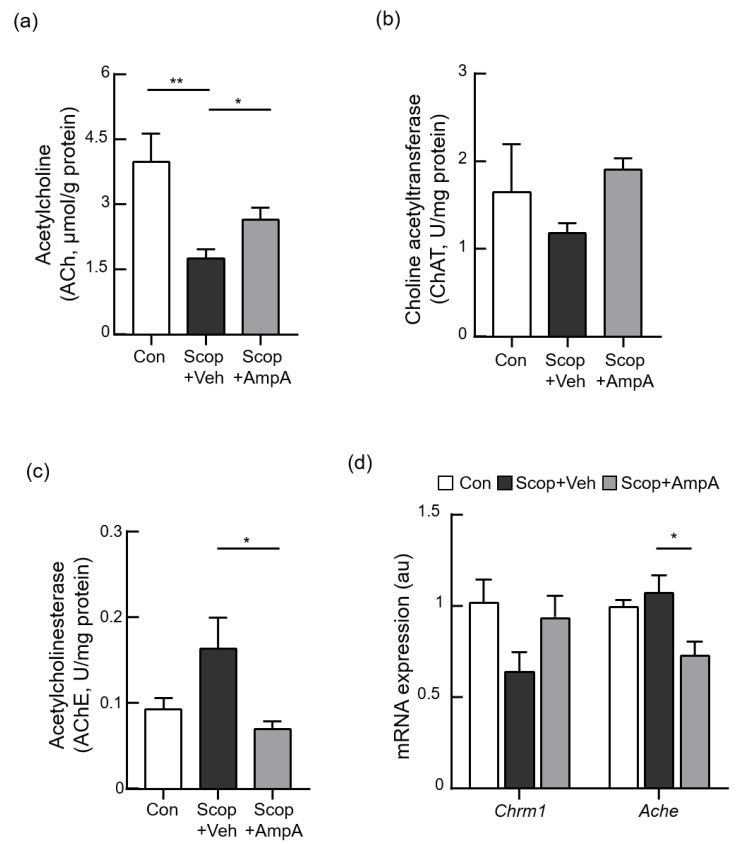
Inhibitory effect of ampelopsin A against scopolamine-induced cholinergic dysfunction. Male C57BL/6J mice (10 weeks old) were given 0.5 µL ampelopsin A (10 ng/µL, three times a week) or the same volume of the vehicle (PBS) via the third-ventricle of the brain for one month. Mice were sacrificed and hippocampi were isolated for measurements of cholinergic parameters and mRNA expression. 30 min before the mice sacrifice, scopolamine (0.8 mg/kg, i.p.) was administered to each group; Scop + Veh (pre-treatment of PBS as a vehicle, scopolamine injection on the experimental day) and Scop + AmpA (pre-treatment of ampelopsin A, scopolamine injection on the experimental day) groups. The control group (Con) was pre-treated with the vehicle into 3 V then was injected PBS (i.p.) instead of scopolamine on the experimental day. (**a**–**c**) Acetylcholine levels and acetylcholinesterase and choline acetyltransferase activities in the hippocampus are shown. (**d**) *Chrm1* and *Ache* mRNA levels determined by real time-PCR. Gene expression was normalized to that of β-actin. Au means the arbitrary units. Values are expressed as means ± SEM (*n* = 4). * *p* < 0.05, ** *p* < 0.01; one-way ANOVA with Dunnett’s multiple comparisons test.

To elucidate the possible molecular mechanisms of ampelopsin A, the levels of acetylcholine and the activities of choline acetyltransferase and acetylcholinesterase that are involved in the acetylcholine metabolism were measured. The hippocampi of mice given scopolamine significantly decreased acetylcholine (ACh) contents and the levels of choline acetyltransferase (ChAT) were reduced by the scopolamine injection. These levels of the ACh and ChAT were recovered in the Scop+AmpA groups (Figure 5a, F_(2,9)_ = 4.602, *p* < 0.05 and Figure 5b, F_(2,9)_ = 1.308). In contrast, the levels of acetylcholinesterase (AChE) activities were increased in the Scop + Veh groups but significantly decreased in the Scop + AmpA groups (Figure 5c, F_(2,9)_ = 4.602, *p* < 0.05). We also measured gene expressions of muscarinic acetylcholine receptor (*Chrm1*, F_(2,9)_ = 2.935) and the acetylcholinesterase (*Ache*, F_(2,10)_ = 6.650, *p* < 0.05). These genes were also changed by the central administration of ampelopsin A (Figure 5d).

### 3.5. Administration of Amplopsin A Elevates BDNF-Related Signaling in the Hippocampus

**Figure 6:**
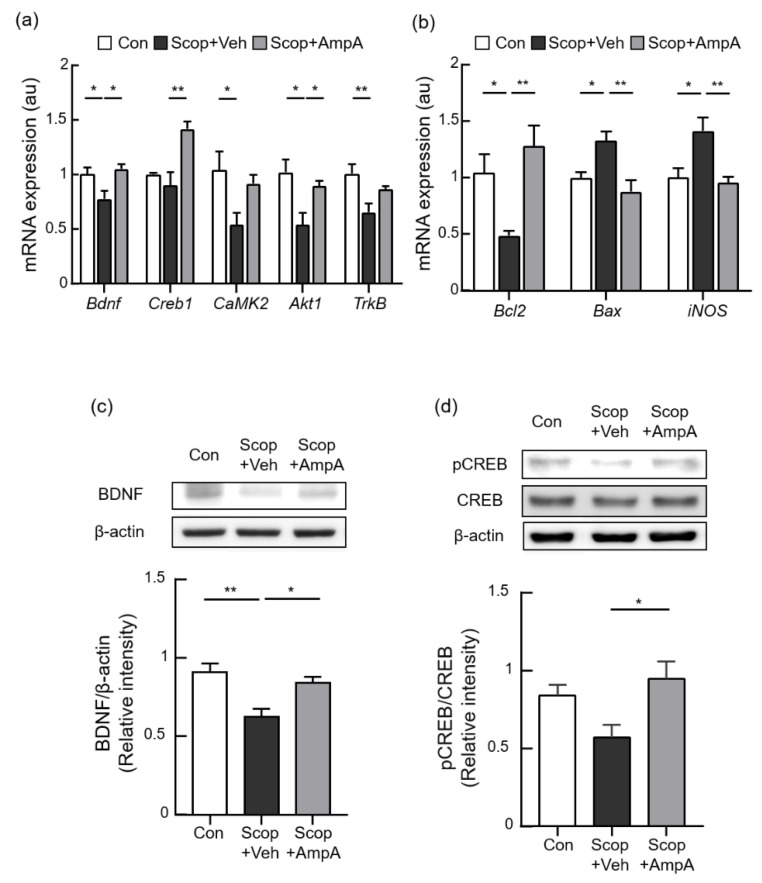
Increase of BDNF-related and anti-apoptotic signaling by central administration of ampelopsin A. Male C57BL/6J mice (10 weeks old) were given 0.5 µL ampelopsin A (10 ng/µL, three times a week) or the same volume of the vehicle (PBS) via the third-ventricle of the brain for one month. Mice were sacrificed and hippocampi were isolated for measurements of mRNA and protein expression. 30 min before the mice sacrifice, scopolamine (0.8 mg/kg, i.p.) was administered to each group; Scop + Veh (pre-treatment of PBS as a vehicle, scopolamine injection on the experimental day) and Scop + AmpA (pre-treatment of ampelopsin A, scopolamine injection on the experimental day) groups. The control group (Con) was pre-treated with the vehicle into 3 V then was injected PBS (i.p.) instead of scopolamine on the experimental day. (**a**) Alterations in the expression of *Bdnf*, *Creb1*, *CaMK2*, *Akt*, and *Trkb* were determined by real time-PCR (*n* = 4). (**b**) *Bcl2*, *Bax*, and *iNOS* mRNA levels determined by real time-PCR (*n* = 4–5). Gene expression was normalized to that of β-actin. Au means the arbitrary units. (**c**) Quantification of BDNF/β-actin and (**d**) phosphorylated CREB/CREB intensity (*n* = 4). Values are expressed as means ± SEM. * *p* < 0.05, ** *p* < 0.01; one-way ANOVA with Dunnett’s multiple comparisons test.

To further elucidate the underlying molecular mechanisms of ampelopsin A, the mRNA and protein expression of CREB/BDNF-related signaling were determined. The CREB1 (F_(2,9)_ = 11.30, *p* < 0.001), BDNF (F_(2,9)_ = 5.912, *p* < 0.05), CaMK2 (F_(2,9)_ = 4.285, *p* < 0.05), Akt (F_(2,9)_ = 6.626, *p* < 0.05), and TrkB (F_(2,10)_ = 7.323, *p* < 0.05) mRNA levels were significantly down-regulated by the Scop + Veh group compared with the control group but were up-regulated in the Scop + AmpA group (Figure 6a). Consistently, scopolamine injection decreased protein levels of BDNF and phosphorylation of CREB in the hippocampus, and the administration of ampelopsin A effectively increased BDNF and pCREB protein levels compared with the administration of scopolamine (Figure 6c, F_(2,9)_ = 8.8, *p* < 0.01 and Figure 5d, F_(2,9)_ = 4.772, *p* < 0.05).

### 3.6. Antioxidant and Anti-Apoptotic Effects on the Hippocampus by Ampelopsin A

Ampelopsin has been known to have antioxidant and anti-apoptotic activities [34,35]. We examined whether central administration of ampelopsin A is responsible for antioxidant and anti-apoptotic effects. The Scop + Veh group significantly increased the mRNA levels of iNOS compared with the control group. This increase was attenuated when ampelopsin A was administrated (Figure 6b, F_(2,12)_ = 7.658, *p* < 0.01). We measured pro-apoptotic and anti-apoptotic effects by the treatments of ampelopsin A. The Scop + Veh group showed a significant increase of Bax (F_(2,12)_ = 7.658, *p* < 0.01) as a pro-apoptotic marker, then it was attenuated by the treatment of ampelopsin A. In contrast, anti-apoptotic Bcl-2 expression altered its expression (F_(2,10)_ = 6.662, *p* < 0.05) then was restored by the treatments of ampelopsin A. The Administration of ampelopsin A recovered apoptotic gene expression in scopolamine-injected mice. These antioxidant and anti-apoptotic effects also support neuroprotective effects of ampelopsin A administration.

## 4. Discussion

Resveratrol (3,5,4-trihydroxystilbene) is a naturally occurring polyphenol that has attracted the attention of many chemists and pharmacologists due to its diverse biological activities such as chemopreventive, antimicrobial, antioxidant, and anti-inflammatory actions [36,37,38,39]. Grapevine is known as an important source of resveratrol and many resveratrol derivatives [40]. The previous other studies showed that extracts, resveratrols, from *V. vinifera* stembark protected the brain cell dysfunction by inhibiting the aggregation of amyloid-β and against α-synuclein cytotoxicity [18,19,20]. Among these extracts, a dimer of resveratrol from *V. vinifera*, ampelopsin A, exhibited more potent anti-amyloidogenic activity than the others [24]. However, it was questionable whether ampelopsin A works on cognitive function for neuroprotective activities in the animal models. Based on the current study, it is quite clear that the brain administration (3 V) of ampleopsin A significantly improved cognitive behaviors, enhanced synaptic transmission, and the cholinergic system in scopolamine-induced memory dysfunction. The underlying mechanisms include but are not limited to the broad up-regulation of genes associated with CREB-BDNF signaling pathways.

In this study, we administrated the relatively low dose of ampelopsin A (10 ng) into the mice brain compared to other references’ uses (µg or mg) [41,42]. The brain responded to this low dose of ampelopsin A to initiate neuroprotective effects in cognition and memory. In addition, we found that chronic administration of ampelopsin A efficiently improved cognition and memory functions whereas acute administration of ampelopsin A did not improve these functions in the experimental animals (data not shown). Usually, chronic treatments were considered as over 10 days to 12 weeks [41,43,44], and similar central treatments for one month showed increases in memory functions [45]. To do so, chronic (a month) and low-dose treatments of ampelopsin A contribute to a change of cognitive and memory abilities.

Cholinesterase (ChE) contributes to the short half-life of released ACh, and it terminates cholinergic neurotransmission by the hydrolysis of ACh in turn. The inhibition of ChEs expression slows down the breakdown of ACh, thereby prolonging ACh presence at synaptic cleft to stimulate their muscarinic receptors. Based on these facts, two major ChEs, AChE and butyrylcholinesterase (BuChE), have been potential targets in AD therapy [46,47]. BuChE is considered to play supportive role in the brain because AChE predominates over BuChE activity [48]. BuChE is also distributed in the hippocampus, but at lower levels than AChE which is mainly located in the synaptic cleft and synaptic membranes in normal status [49,50]. Since our study is the first study suggesting the ampelopsin A as a ChE inhibitor, we focused on the inhibitory activity of AChE as the hippocampal cholinergic mediation. As BuChE has brought much attention compensating for the action of AChE in cognitive impairment, further studies will establish the detailed influence of stilbenoids on BuChE for a beneficial feature in AD treatment [51,52,53].

Scopolamine, a muscarinic acetylcholine receptor antagonist, is a commonly used chemical that impairs learning and memory in animal models. Scopolamine-induced deficits in a battery of cognitive function are important for comparison of sensitivity and specificity to find therapeutic candidates for neurodegenerative diseases [54,55]. The exact mechanism of scopolamine action to ACh, ChAT, and ChE remains poorly understood. Since scopolamine has been used in the standard cognitive impairment model, there were a lot of literatures to show that the effects of scopolamine treatment can induce cognitive deficit through decreasing ACh contents and ChAT activities while increasing AChE activities in the hippocampus [10,56,57]. The stilbenoids, including ampelopsin A, have been studied as the potent AChE inhibitors for developing AD-targeting drugs [21,22,23]. Ampelopsin A may be considered as an AD-targeting drug by its anti-AChE activity [24]. In addition, this cholinergic system contributes to neurogenesis in the hippocampus via the CREB/BDNF signaling which is responsible for long-term memory formation [58,59]. Based on our study, administration of ampelopsin A delayed deficit of cholinergic cognitive memory and ameliorated long-term memory by restoring CREB/BDNF signaling. Therefore, ampelopsin A might be considered a strong candidate for treating AD to recover acetylcholine cascades in the hippocampus with reduced symptoms [10,60,61].

The avoidance reaction of an experimental mouse is important for the acquisition of extinction memory. In the passive avoidance test, the animal learns to avoid an unpleasant stimulus by hindering locomotion and investigation [62]. Additionally, treatments with an anti-BDNF antibody or BDNF antisense mRNA produce memory dysfunction in concurrence with a loss of LTP and ERK signaling [62,63,64]. To do so, the hippocampal BDNF-TrkB signaling is required for the acquisition and consolidation of conditioned fear [65,66]. In addition, hippocampus-specific deletion of BDNF lessens fear extinction, while hippocampal BDNF accelerates the acquisition of extinction memory [67]. BDNF is one of the crucial factors to form fear extinction memory [62,67,68]. Our study showed that ampelopsin A significantly increased the avoidance reaction of experimental mice and up-regulated hippocampal BDNF/CREB cascades, including BDNF, CREB1, CaMK2, Akt, and TrkB. Although cognition and memory functional mechanisms mediated by each gene may be different, these genes are closely associated with avoiding aversive stimulus in the memory regions. We assumed that ampleopsin A may stimulate BDNF-CREB signaling in the hippocampus for increases of memory function although more detailed experiments are necessary. In addition, scopolamine induces an increase in neuro-inflammation (iNOS) and apoptosis (Bax) while it inhibits anti-apoptotic factors (Bcl-2) [69]. Our study showed that ampelopsin A has neuroprotective effects by reversing molecular and cell damages released from neuroinflammation and apoptosis.

Long-term potentiation (LTP) represents a long-lasting increase in the efficacy of excitatory synaptic transmission, and it is widely used to measure a cellular mechanism of learning and memory in the brain [30,31]. Among all neurotransmitters and trophic factors, BDNF and glutamate are mostly related to memory function [62]. BDNF directly works on depolarizing neurons by enhancing glutamatergic transmission for inducing phosphorylation of NMDA receptor through its TrkB receptors [70]. The BDNF utilizes positive regulations on LTP in memory formation at the cellular level. In addition, impairment of LTP in mutant mice lacking BDNF was rescued by recombinant BDNF application [71]. The endogenous BDNF is necessary for LTP formation which comes out from presynaptic neurons and BDNF-dependent LTP formation is responsible for protein synthesis [71,72]. The BDNF mediates the translation of protein synthesis via several intracellular signaling pathways including Akt and PI3K, kinases involved in cell growth, survival, differentiation, and intracellular trafficking. Our study showed that the chronic administration of ampelopsin A into the brain rescues the scopolamine-induced deficit in hippocampal LTP through BDNF activation. The recovered capability of LTP in the brain is important for brain protection in neurodegenerative diseases. Chronic administration of ampelopsin A might be considered a therapy for neurodegenerative disease by recovering functional LTP in the brain.

## 5. Conclusions

The central administration of ampelopsin A ameliorates scopolamine-induced cognitive impairment in the brain. These effects of ampelopsin A might be related to restored LTP through BDNF activation.

## Data Availability

All datasets of this study are generated in the article.
